# A qualitative study of nursing student experiences of clinical practice

**DOI:** 10.1186/1472-6955-4-6

**Published:** 2005-11-09

**Authors:** Farkhondeh Sharif, Sara Masoumi

**Affiliations:** 1 Psychiatric Nursing Department, Fatemeh (P.B.U.H) College of Nursing and Midwifery Shiraz University of Medical Sciences, Zand BlvD, Shiraz, Iran; 2 English Department, Shiraz University, Shiraz, Iran

## Abstract

**Background:**

Nursing student's experiences of their clinical practice provide greater insight to develop an effective clinical teaching strategy in nursing education. The main objective of this study was to investigate student nurses' experience about their clinical practice.

**Methods:**

Focus groups were used to obtain students' opinion and experiences about their clinical practice. 90 baccalaureate nursing students at Shiraz University of Medical Sciences (Faculty of Nursing and Midwifery) were selected randomly from two hundred students and were arranged in 9 groups of ten students. To analyze the data the method used to code and categories focus group data were adapted from approaches to qualitative data analysis.

**Results:**

Four themes emerged from the focus group data. From the students' point of view," initial clinical anxiety", "theory-practice gap"," clinical supervision", professional role", were considered as important factors in clinical experience.

**Conclusion:**

The result of this study showed that nursing students were not satisfied with the clinical component of their education. They experienced anxiety as a result of feeling incompetent and lack of professional nursing skills and knowledge to take care of various patients in the clinical setting.

## Background

Clinical experience has been always an integral part of nursing education. It prepares student nurses to be able of "doing" as well as "knowing" the clinical principles in practice. The clinical practice stimulates students to use their critical thinking skills for problem solving [1]

Awareness of the existence of stress in nursing students by nurse educators and responding to it will help to diminish student nurses experience of stress. [2]

Clinical experience is one of the most anxiety producing components of the nursing program which has been identified by nursing students. In a descriptive correlational study by Beck and Srivastava 94 second, third and fourth year nursing students reported that clinical experience was the most stressful part of the nursing program[3]. Lack of clinical experience, unfamiliar areas, difficult patients, fear of making mistakes and being evaluated by faculty members were expressed by the students as anxiety-producing situations in their initial clinical experience. In study done by Hart and Rotem stressful events for nursing students during clinical practice have been studied. They found that the initial clinical experience was the most anxiety producing part of their clinical experience [4]. The sources of stress during clinical practice have been studied by many researchers [5-10] and [11].

The researcher came to realize that nursing students have a great deal of anxiety when they begin their clinical practice in the second year. It is hoped that an investigation of the student's view on their clinical experience can help to develop an effective clinical teaching strategy in nursing education.

## Methods

A focus group design was used to investigate the nursing student's view about the clinical practice. Focus group involves organized discussion with a selected group of individuals to gain information about their views and experiences of a topic and is particularly suited for obtaining several perspectives about the same topic. Focus groups are widely used as a data collection technique. The purpose of using focus group is to obtain information of a qualitative nature from a predetermined and limited number of people [12,13].

Using focus group in qualitative research concentrates on words and observations to express reality and attempts to describe people in natural situations [14].

The group interview is essentially a qualitative data gathering technique [13]. It can be used at any point in a research program and one of the common uses of it is to obtain general background information about a topic of interest [14].

Focus groups interviews are essential in the evaluation process as part of a need assessment, during a program, at the end of the program or months after the completion of a program to gather perceptions on the outcome of that program [15,16]. Kruegger (1988) stated focus group data can be used before, during and after programs in order to provide valuable data for decision making [12].

The participants from which the sample was drawn consisted of 90 baccalaureate nursing students from two hundred nursing students (30 students from the second year and 30 from the third and 30 from the fourth year) at Shiraz University of Medical Sciences (Faculty of Nursing and Midwifery). The second year nursing students already started their clinical experience. They were arranged in nine groups of ten students. Initially, the topics developed included 9 open-ended questions that were related to their nursing clinical experience. The topics were used to stimulate discussion.

The following topics were used to stimulate discussion regarding clinical experience in the focus groups.

1. How do you feel about being a student in nursing education?

2. How do you feel about nursing in general?

3. Is there any thing about the clinical field that might cause you to feel anxious about it?

4. Would you like to talk about those clinical experiences which you found most anxiety producing?

5. Which clinical experiences did you find enjoyable?

6. What are the best and worst things do you think can happen during the clinical experience?

7. What do nursing students worry about regarding clinical experiences?

8. How do you think clinical experiences can be improved?

9. What is your expectation of clinical experiences?

The first two questions were general questions which were used as ice breakers to stimulate discussion and put participants at ease encouraging them to interact in a normal manner with the facilitator.

### Data analysis

The following steps were undertaken in the focus group data analysis.

1. Immediate debriefing after each focus group with the observer and debriefing notes were made. Debriefing notes included comments about the focus group process and the significance of data

2. Listening to the tape and transcribing the content of the tape

3. Checking the content of the tape with the observer noting and considering any non-verbal behavior. The benefit of transcription and checking the contents with the observer was in picking up the following:

a. Parts of words

b. Non-verbal communication, gestures and behavior...

The researcher facilitated the groups. The observer was a public health graduate who attended all focus groups and helped the researcher by taking notes and observing students' on non-verbal behavior during the focus group sessions. Observer was not known to students and researcher

The methods used to code and categorise focus group data were adapted from approaches to qualitative content analysis discussed by Graneheim and Lundman [17] and focus group data analysis by Stewart and Shamdasani [14] For coding the transcript it was necessary to go through the transcripts line by line and paragraph by paragraph, looking for significant statements and codes according to the topics addressed. The researcher compared the various codes based on differences and similarities and sorted into categories and finally the categories was formulated into a 4 themes.

The researcher was guided to use and three levels of coding [17,18]. Three levels of coding selected as appropriate for coding the data.

Level 1 coding examined the data line by line and making codes which were taken from the language of the subjects who attended the focus groups.

Level 2 coding which is a comparing of coded data with other data and the creation of categories. Categories are simply coded data that seem to cluster together and may result from condensing of level 1 code [17,19].

Level 3 coding which describes the Basic Social Psychological Process which is the title given to the central themes that emerge from the categories.

Table 1 shows the three level codes for one of the theme

**Table 1:** Examples of 3 levels of coding

Level 1 codes (Meaning unit)	Level 2 codes (categories)	Level 3 codes (theme)
• Lack of confidence	* Fear of failure	
• Lack of knowledge	*Feeling incompetent	Initial clinical
• Lack of confidence in the first day	*Feeling under pressure	Anxiety
	*Fear of facing the procedure	
• Being anxious about Starting clinical practice		
• Fear of hospital environment		
• First week anxiety		
• Fear of unknown in the first day		

The documents were submitted to two assessors for validation. This action provides an opportunity to determine the reliability of the coding [14,15]. Following a review of the codes and categories there was agreement on the classification.

### Ethical considerations

The study was conducted after approval has been obtained from Shiraz university vice-chancellor for research and in addition permission to conduct the study was obtained from Dean of the Faculty of Nursing and Midwifery. All participants were informed of the objective and design of the study and a written consent received from the participants for interviews and they were free to leave focus group if they wish.

## Results

Most of the students were females (%94) and single (% 86) with age between 18–25.

The qualitative analysis led to the emergence of the four themes from the focus group data. From the students' point of view," initial clinical anxiety", "theory-practice gap", clinical supervision"," professional role", was considered as important factors in clinical experience.

### Initial clinical anxiety

This theme emerged from all focus group discussion where students described the difficulties experienced at the beginning of placement. Almost all of the students had identified feeling anxious in their initial clinical placement. Worrying about giving the wrong information to the patient was one of the issues brought up by students.

One of the students said:

On the first day I was so anxious about giving the wrong information to the patient. I remember one of the patients asked me what my diagnosis is. ' I said 'I do not know', she said 'you do not know? How can you look after me if you do not know what my diagnosis is?'

From all the focus group sessions, the students stated that the first month of their training in clinical placement was anxiety producing for them.

One of the students expressed:

*The most stressful situation is when we make the next step. I mean*...*clinical placement and we don't have enough clinical experience to accomplish the task, and do our nursing duties*.

Almost all of the fourth year students in the focus group sessions felt that their stress reduced as their training and experience progressed.

Another cause of student's anxiety in initial clinical experience was the students' concern about the possibility of harming a patient through their lack of knowledge in the second year.

One of the students reported:

*In the first day of clinical placement two patients were assigned to me. One of them had IV fluid. When I introduced myself to her, I noticed her IV was running out. I was really scared and I did not know what to do and I called my instructor*.

Fear of failure and making mistakes concerning nursing procedures was expressed by another student. She said:

*I was so anxious when I had to change the colostomy dressing of my 24 years old patient. It took me 45 minutes to change the dressing. I went ten times to the clinic to bring the stuff. My heart rate was increasing and my hand was shaking. I was very embarrassed in front of my patient and instructor. I will never forget that day*.

Sellek researched anxiety-creating incidents for nursing students. He suggested that the ward is the best place to learn but very few of the learner's needs are met in this setting. Incidents such as evaluation by others on initial clinical experience and total patient care, as well as interpersonal relations with staff, quality of care and procedures are anxiety producing [11].

### Theory-practice gap

The category theory-practice gap emerged from all focus discussion where almost every student in the focus group sessions described in some way the lack of integration of theory into clinical practice.

*I have learnt so many things in the class, but there is not much more chance to do them in actual settings*.

Another student mentioned:

*When I just learned theory for example about a disease such as diabetic mellitus and then I go on the ward and see the real patient with diabetic mellitus, I relate it back to what I learned in class and that way it will remain in my mind. It is not happen sometimes*.

The literature suggests that there is a gap between theory and practice. It has been identified by Allmark and Tolly [20,21]. The development of practice theory, theory which is developed from practice, for practice, is one way of reducing the theory-practice gap [21]. Rolfe suggests that by reconsidering the relationship between theory and practise the gap can be closed. He suggests facilitating reflection on the realities of clinical life by nursing theorists will reduce the theory-practice gap. The theory- practice gap is felt most acutely by student nurses. They find themselves torn between the demands of their tutor and practising nurses in real clinical situations. They were faced with different real clinical situations and are unable to generalise from what they learnt in theory [22].

### Clinical supervision

Clinical supervision is recognised as a developmental opportunity to develop clinical leadership. Working with the practitioners through the milieu of clinical supervision is a powerful way of enabling them to realize desirable practice [23]. Clinical nursing supervision is an ongoing systematic process that encourages and supports improved professional practice. According to Berggren and Severinsson the clinical nurse supervisors' ethical value system is involved in her/his process of decision making. [24,25]

Clinical Supervision by Head Nurse (Nursing Unit Manager) and Staff Nurses was another issue discussed by the students in the focus group sessions. One of the students said:

Sometimes we are taught mostly by the Head Nurse or other Nursing staff. The ward staff are not concerned about what students learn, they are busy with their duties and they are unable to have both an educational and a service role

Another student added:

Some of the nursing staff have good interaction with nursing students and they are interested in helping students in the clinical placement but they are not aware of the skills and strategies which are necessary in clinical education and are not prepared for their role to act as an instructor in the clinical placement

The students mostly mentioned their instructor's role as an evaluative person. The majority of students had the perception that their instructors have a more evaluative role than a teaching role.

The literature suggests that the clinical nurse supervisors should expressed their existence as a role model for the supervisees [24]

### Professional role

One view that was frequently expressed by student nurses in the focus group sessions was that students often thought that their work was 'not really professional nursing' they were confused by what they had learned in the faculty and what in reality was expected of them in practice.

*We just do basic nursing care, very basic*. ...*You know*...*giving bed baths, keeping patients clean and making their beds. Anyone can do it. We spend four years studying nursing but we do not feel we are doing a professional job*.

The role of the professional nurse and nursing auxiliaries was another issue discussed by one of the students:

*The role of auxiliaries such as registered practical nurse and Nurses Aids are the same as the role of the professional nurse. We spend four years and we have learned that nursing is a professional job and it requires training and skills and knowledge, but when we see that Nurses Aids are doing the same things, it can not be considered a professional job*.

## Discussion

The result of student's views toward clinical experience showed that they were not satisfied with the clinical component of their education. Four themes of concern for students were 'initial clinical anxiety', 'theory-practice gap', 'clinical supervision', and 'professional role'.

The nursing students clearly identified that the initial clinical experience is very stressful for them. Students in the second year experienced more anxiety compared with third and fourth year students. This was similar to the finding of Bell and Ruth who found that nursing students have a higher level of anxiety in second year [26,27]. Neary identified three main categories of concern for students which are the fear of doing harm to patients, the sense of not belonging to the nursing team and of not being fully competent on registration [28] which are similar to what our students mentioned in the focus group discussions. Jinks and Patmon also found that students felt they had an insufficiency in clinical skills upon completion of pre-registration program [29].

Initial clinical experience was the most anxiety producing part of student clinical experience. In this study fear of making mistake (fear of failure) and being evaluated by faculty members were expressed by the students as anxiety-producing situations in their initial clinical experience. This finding is supported by Hart and Rotem [4] and Stephens [30]. Developing confidence is an important component of clinical nursing practice [31]. Development of confidence should be facilitated by the process of nursing education; as a result students become competent and confident. Differences between actual and expected behaviour in the clinical placement creates conflicts in nursing students. Nursing students receive instructions which are different to what they have been taught in the classroom. Students feel anxious and this anxiety has effect on their performance [32]. The existence of theory-practice gap in nursing has been an issue of concern for many years as it has been shown to delay student learning. All the students in this study clearly demonstrated that there is a gap between theory and practice. This finding is supported by other studies such as Ferguson and Jinks [33] and Hewison and Wildman [34] and Bjork [35]. Discrepancy between theory and practice has long been a source of concern to teachers, practitioners and learners. It deeply rooted in the history of nurse education. Theory-practice gap has been recognised for over 50 years in nursing. This issue is said to have caused the movement of nurse education into higher education sector [34].

Clinical supervision was one of the main themes in this study. According to participant, instructor role in assisting student nurses to reach professional excellence is very important. In this study, the majority of students had the perception that their instructors have a more evaluative role than a teaching role. About half of the students mentioned that some of the head Nurse (Nursing Unit Manager) and Staff Nurses are very good in supervising us in the clinical area. The clinical instructor or mentors can play an important role in student nurses' self-confidence, promote role socialization, and encourage independence which leads to clinical competency [36]. A supportive and socialising role was identified by the students as the mentor's function. This finding is similar to the finding of Earnshaw [37]. According to Begat and Severinsson supporting nurses by clinical nurse specialist reported that they may have a positive effect on their perceptions of well-being and less anxiety and physical symptoms [25].

The students identified factors that influence their professional socialisation. Professional role and hierarchy of occupation were factors which were frequently expressed by the students. Self-evaluation of professional knowledge, values and skills contribute to the professional's self-concept [38]. The professional role encompasses skills, knowledge and behaviour learned through professional socialisation [39]. The acquisition of career attitudes, values and motives which are held by society are important stages in the socialisation process [40]. According to Corwin autonomy, independence, decision-making and innovation are achieved through professional self-concept 41. Lengacher (1994) discussed the importance of faculty staff in the socialisation process of students and in preparing them for reality in practice. Maintenance and/or nurturance of the student's self-esteem play an important role for facilitation of socialisation process 42.

One view that was expressed by second and third year student nurses in the focus group sessions was that students often thought that their work was 'not really professional nursing' they were confused by what they had learned in the faculty and what in reality was expected of them in practice.

## Conclusion

The finding of this study and the literature support the need to rethink about the clinical skills training in nursing education. It is clear that all themes mentioned by the students play an important role in student learning and nursing education in general. There were some similarities between the results of this study with other reported studies and confirmed that some of the factors are universal in nursing education. Nursing students expressed their views and mentioned their worry about the initial clinical anxiety, theory-practice gap, professional role and clinical supervision. They mentioned that integration of both theory and practice with good clinical supervision enabling them to feel that they are enough competent to take care of the patients. The result of this study would help us as educators to design strategies for more effective clinical teaching. The results of this study should be considered by nursing education and nursing practice professionals. Faculties of nursing need to be concerned about solving student problems in education and clinical practice. The findings support the need for Faculty of Nursing to plan nursing curriculum in a way that nursing students be involved actively in their education.

## Competing interests

The author(s) declare that they no competing interests.

## Authors' contributions

FSH: Initiation and design of the research, focus groups conduction, data collection, analysis and writing the paper, SM: Editorial revision of paper

## Pre-publication history

The pre-publication history for this paper can be accessed here:


